# Smoking and lateral hinge fracture are associated with delayed union at 6 months in open wedge distal tibial tuberosity osteotomy

**DOI:** 10.1002/jeo2.70829

**Published:** 2026-07-07

**Authors:** Atsuki Tanaka, Ryo Okada, Yuta Nakanishi, Takahiro Yamashita, Shohei Sano, Daisuke Araki

**Affiliations:** ^1^ Department of Orthopaedic Surgery Hyogo Prefectural Rehabilitation Central Hospital Kobe Japan; ^2^ Department of Orthopaedic Surgery Kobe University Graduate School of Medicine Kobe Japan; ^3^ Sports Medicine Center Hyogo Prefectural Rehabilitation Central Hospital Kobe Japan; ^4^ Fowler Kennedy Sport Medicine Clinic University of Western Ontario London Ontario Canada

**Keywords:** computed tomography evaluation, distal flange union, high tibial osteotomy, posterior cortex union

## Abstract

**Purpose:**

This study aimed to identify risk factors for delayed union 6 months after open wedge distal tibial tuberosity osteotomy (owDTO) using computed tomography (CT) and to evaluate the association between distal flange and posterior cortex union.

**Methods:**

This retrospective study was conducted on all patients who underwent owDTO. The exclusion criteria were failure to undergo a 6‐month postoperative follow‐up, absence of CT evaluation, simultaneous anterior cruciate ligament reconstruction, double‐level osteotomy and cases requiring multiple surgical debridements for deep infection. Bone union rates at the flange and posterior cortex were evaluated using CT images obtained 6 months postoperatively. Bone union was determined using zone classification from a previous report. Various factors influencing bone union were examined. Statistical significance was set at *p* < 0.05.

**Results:**

Of the 122 knees reviewed, a total of 79 knees from 77 patients (43 males and 34 females; age, 55 ± 10 years) were analysed after the exclusion of 43 knees. Twenty‐two patients were current smokers. The body mass index was 25.1 ± 3.8 kg/m^2^. Bone union at 6 months was observed in the posterior cortex, distal flange and proximal flange in 55 (69.6%), 67 (84.8%) and 76 (96.2%) knees, respectively. Lateral hinge fracture (LHF) occurred in 20 knees. The posterior cortex union rate was significantly higher in the distal flange union group than in the delayed union group (77.6% vs. 25.0%, *p* < 0.01). LHF was significantly associated with posterior cortex delayed union (*p* = 0.03). Smoking was significantly associated with distal flange delayed union (*p* = 0.02).

**Conclusions:**

Smoking and LHF were associated with delayed union at 6 months in owDTO. Distal flange union was significantly associated with posterior cortex union. These findings may assist surgeons in identifying patients at risk for delayed union after owDTO and may contribute to improved postoperative management.

**Level of Evidence:**

Level IV, retrospective study.

## Graphical abstract

Smoking and lateral hinge fracture were associated with delayed union at 6 months in open wedge distal tibial tuberosity osteotomy. Distal flange union was significantly associated with posterior cortical union.

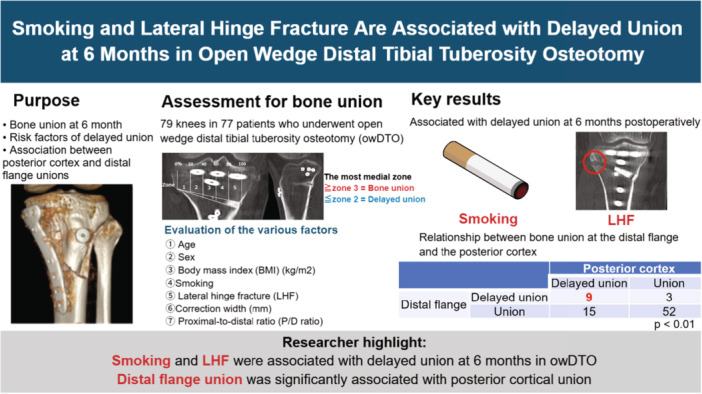

**Abbreviations**
BMI: body mass index
CI: confidence interval
CT: computed tomography
D: distal
HTO: high tibial osteotomy
ICC: intraclass correlation coefficient
KL: Kellgren–Lawrence
LHF: lateral hinge fracture
MMPRT: medial meniscus posterior root tear
OR: odds ratio
owDTO: open wedge distal tibial tuberosity osteotomy
P: posterior
P/D ratio: proximal‐to‐distal ratio
PTFJ: proximal tibiofibular joint
%MA: percent mechanical axis

## INTRODUCTION

High tibial osteotomy (HTO) was established as a joint‐preserving treatment for varus knee osteoarthritis [10, 27]. Open wedge HTO does not require fibular osteotomy and allows relatively easy adjustment of the correction angle, in contrast to conventional closed‐wedge HTO. Consequently, open wedge HTO has developed with the widespread use of locking plates [30, 33] and has consistently shown good clinical outcomes. However, a disadvantage of open wedge HTO is that distal displacement of the tibial tubercle may cause postoperative patella baja, thereby increasing patellofemoral joint pressure and potentially leading to subsequent cartilage injury [14].

**Figure 1:**
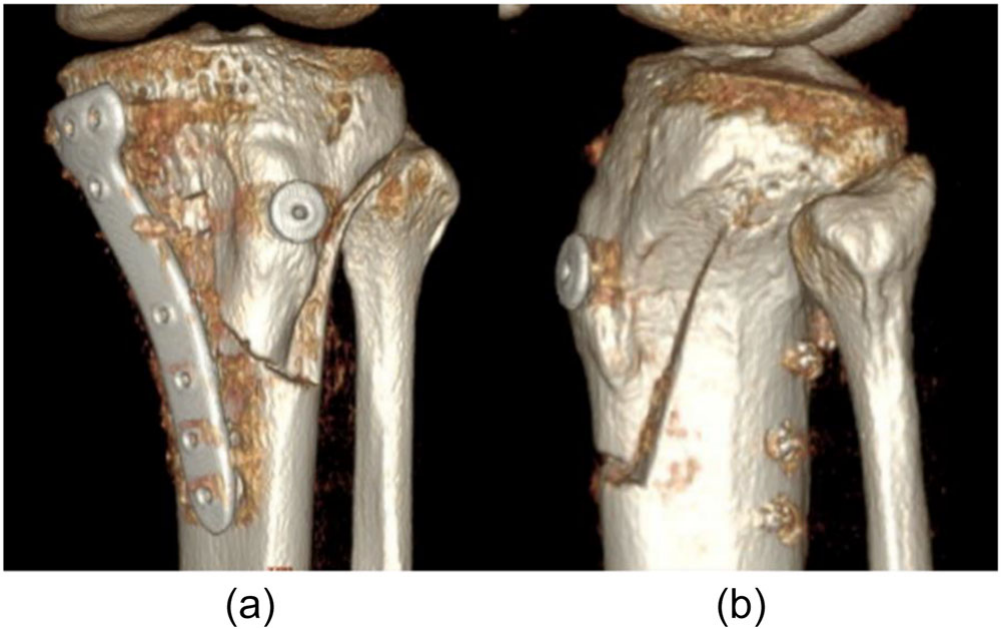
Postoperative two‐week image of open wedge distal tibial tuberosity osteotomy (owDTO). (a) Frontal view. (b) Lateral view. In owDTO, the tibial tuberosity remains attached to the proximal bone fragment. A screw is inserted from the distal tuberosity into the tibial shaft.

Open wedge distal tibial tuberosity osteotomy (owDTO) has gained attention in recent years as a technique to avoid patella baja and subsequent increase in patellofemoral joint pressure [14, 18]. owDTO leaves the tibial tuberosity attached to the proximal bone fragment, allowing the procedure to prevent excessive contact pressure on the patellofemoral joint (Figure 1) [2, 32]. Therefore, owDTO does not exacerbate patellofemoral osteoarthritis [11, 22]. The clinical outcomes have also been favourable, indicating that owDTO is an effective treatment for varus osteoarthritis with patellofemoral osteoarthritis [7].

Complications of owDTO have been reported, including lateral hinge fracture (LHF), tibial tuberosity fracture, delayed union and nonunion [1, 24, 32]. Delayed union is a complication that should be avoided because it increases the risk of prolonged pain, nonunion and implant failure [3, 4, 9, 19, 26]. Risk factors such as smoking, obesity, a larger opening gap, unstable hinge fractures and anterior plate placement have been identified for delayed union and nonunion in open wedge HTO [3, 4, 9, 19]. However, only a limited number of reports have investigated risk factors for delayed union in owDTO, and the available evidence remains insufficient and controversial [23, 25, 26].

Therefore, the primary aim of this study was to investigate risk factors for delayed union after owDTO using computed tomography (CT) at 6 months postoperatively. The secondary aim was to evaluate the association between distal flange union and posterior cortex union. It was hypothesized that LHF would be associated with delayed union, and that distal flange union would be associated with posterior cortex union.

## METHODS

### Patient selection

This retrospective study was conducted in accordance with the Declaration of Helsinki and was approved by the institutional review board (IRB No. 2306) and conducted using an opt‐out approach. All patients had also provided written consent, including permission for the use of their clinical data for research purposes. Patients who underwent owDTO between May 2021 and January 2023 at the study institution were reviewed. At a single institution, owDTO was routinely performed instead of conventional open wedge HTO during the study period. The indication for owDTO was symptomatic medial compartment pain with malalignment. A total of 122 knees in 111 patients were screened for eligibility. Thirty‐two knees had medial meniscus posterior root tears (MMPRTs), and 62 knees had other reparable meniscus tears. The exclusion criteria were failure to undergo a 6‐month postoperative follow‐up, absence of CT evaluation at 6 months postoperatively, simultaneous anterior cruciate ligament reconstruction, double‐level osteotomy and cases requiring multiple surgical debridements for postoperative deep infection.

On the day of admission, the body mass index (BMI) was calculated, and smoking status was defined as current smoking at the time of surgery based on the medical record. Radiographic severity of osteoarthritis was assessed using the Kellgren–Lawrence (KL) grading system (Grades 0–4). Preoperative and postoperative lower limb alignment was assessed using supine long‐leg radiographs. The percent mechanical axis (%MA) was defined as the weight‐bearing line extending from the centre of the femoral head to the centre of the ankle joint, and its value was expressed as the percentage across the tibial plateau from the medial edge.

### Surgical procedures

The surgical technique for owDTO, as described by Akiyama et al., was followed [2]. The hinge position was decided within the proximal tibiofibular joint (PTFJ) using fluoroscopy [32]. The flange area was cut using arc osteotomy to secure a larger contact area for each fragment. The radius of the arc from the hinge position was set to 60 mm for males and 55 mm for females. A Medial HTO Plate System (OSferion Biomaterials Corp.) and a HOLLYX CCS (HOLLYX) were used as implants. A HOLLYX CCS was used to fix the distal tuberosity to the tibial shaft. β‐tricalcium phosphate (OSferion Biomaterials Corp.) was inserted to fill the wedge gap to promote bone healing. The correction target was the lateral tibial intercondylar eminence [12]. In cases requiring a large correction in which the predicted postoperative medial proximal tibial angle would exceed 94°, double‐level osteotomy was selected instead of isolated owDTO. Regarding meniscus tears, MMPRTs were treated using transtibial pull‐out repair, whereas other reparable meniscal tears were treated using arthroscopic meniscal repair techniques. For postoperative therapy, immobilization with knee bracing and non‐weight bearing was conducted until postoperative Day 7, and range‐of‐motion training and partial weight bearing were initiated on postoperative Day 8. Weight bearing was gradually increased over the following weeks, with full weight bearing permitted at postoperative Week 4. If an obvious LHF was detected during surgery, weight bearing was delayed by one week compared to the standard protocol. Jogging was permitted at 3 months postoperatively, and if bone union was confirmed by CT evaluation at 6 months postoperatively, return to sports activities such as running was allowed.

### CT evaluation

**Figure 2:**
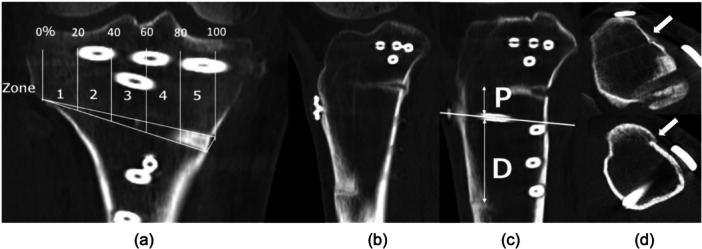
Assessment for bone union. (a) The osteotomy gap was divided into five zones on the coronal plane of the slice within proximal tibiofibular joint (PTFJ) and numbered as Zones 1–5 from the lateral side. (b) Bone union of the posterior cortex was evaluated in each of the five zones in the sagittal plane. The most medial zone achieving bone union was assessed, and bone union was considered if union was observed in Zone 3 or beyond. (c) Flange was divided into proximal (P) and distal (D) sections relative to the screw. (d) Bone union was evaluated on the axial plane.

Bone union of the posterior cortex and flange was assessed using a CT scanner (Revolution EVO; GE Healthcare) at 6 months postoperatively with 0.625 mm thick slices. Multiplanar reconstructed images with a slice thickness of 2 mm were used for evaluation. CT scans were routinely performed at 2 weeks and 6 months postoperatively, as they allow a more detailed assessment of bone union and LHF than radiography [15, 16]. Bone union in the posterior cortex was evaluated using the method proposed by Kobayashi et al. [16]. Kobayashi et al. conducted a CT evaluation based on Brosset et al.'s radiological bone union assessment method [5]. The osteotomy gap was divided into five zones on the coronal plane of the slice within the PTFJ and numbered 1–5 from the lateral side (Figure 2a). Bone union in the posterior cortex was evaluated in each of the five zones in the sagittal plane (Figure 2b). The most medial zone achieving bone union was assessed, and bone union was considered present if union was observed in Zone 3 or beyond. In contrast, the flanges were divided into proximal (P) and distal (D) sections relative to the screw, and bone union was evaluated in the axial plane (Figure 2c,d). The relationship between bone union at the distal flange and the posterior cortex was investigated because bone union at the distal flange may reflect micromotion at the osteotomy site and could therefore be associated with posterior cortex union. The distances from the screw to the proximal and distal ends of the flange were measured. The P and D lengths were measured, and the proximal‐to‐distal ratio (P/D ratio) was calculated.

Various factors influencing bone union, including age, sex, BMI, smoking status, LHF, correction width and P/D ratio, were examined. LHF was assessed using these CT images and classified according to Takeuchi's classification [31]. This classification divides LHF into three groups: Type I, extending along the osteotomy line and remaining proximal to or within the PTFJ; Type II, extending distally into the PTFJ and Type III, involving the lateral tibial plateau.

### Statistical evaluation

All statistical analyses were performed using EZR (Jichi Medical University), a graphical user interface for R (R Foundation for Statistical Computing) [13]. EZR is a modified version of R Commander, designed to include statistical functions that are frequently used in biostatistics.

Fisher's exact test was used to analyse the relationship between bone union at the distal flange and the posterior cortex, as well as sex, LHF and smoking status. Student's *t* test was used to compare age, BMI and P/D ratio. The Mann–Whitney *U* test was used to compare correction width. Statistical significance was set at *p* < 0.05. After univariate analyses, variables with *p* < 0.10 and those considered clinically relevant (age, BMI, sex) were included as independent variables in separate multivariate logistic regression analyses, with bone union of the posterior cortex and bone union of the distal flange as the dependent variables.

CT evaluation was independently performed by two reviewers who were fellowship‐trained orthopaedic surgeons. Intraclass correlation coefficients (ICCs) were used to evaluate inter‐ and intra‐rater reliability for bone union evaluation of the posterior cortex on CT images. Intra‐rater reliability was evaluated using 30 randomly selected cases that were reviewed by the same reviewers with an interval of at least 6 months between assessments. ICC values < 0.50 were considered poor, 0.50–0.75 as moderate, 0.75–0.90 as good and >0.90 as excellent reliability [17].

A post hoc power analysis for Student's *t* test was performed using G*Power to compare the posterior cortex union and distal flange union groups. An effect size of 0.80 and an *α* error probability of 0.05 were used. The calculated power values were 0.94 and 0.81, respectively, indicating an adequate sample size [8].

## RESULTS

**Figure 3:**
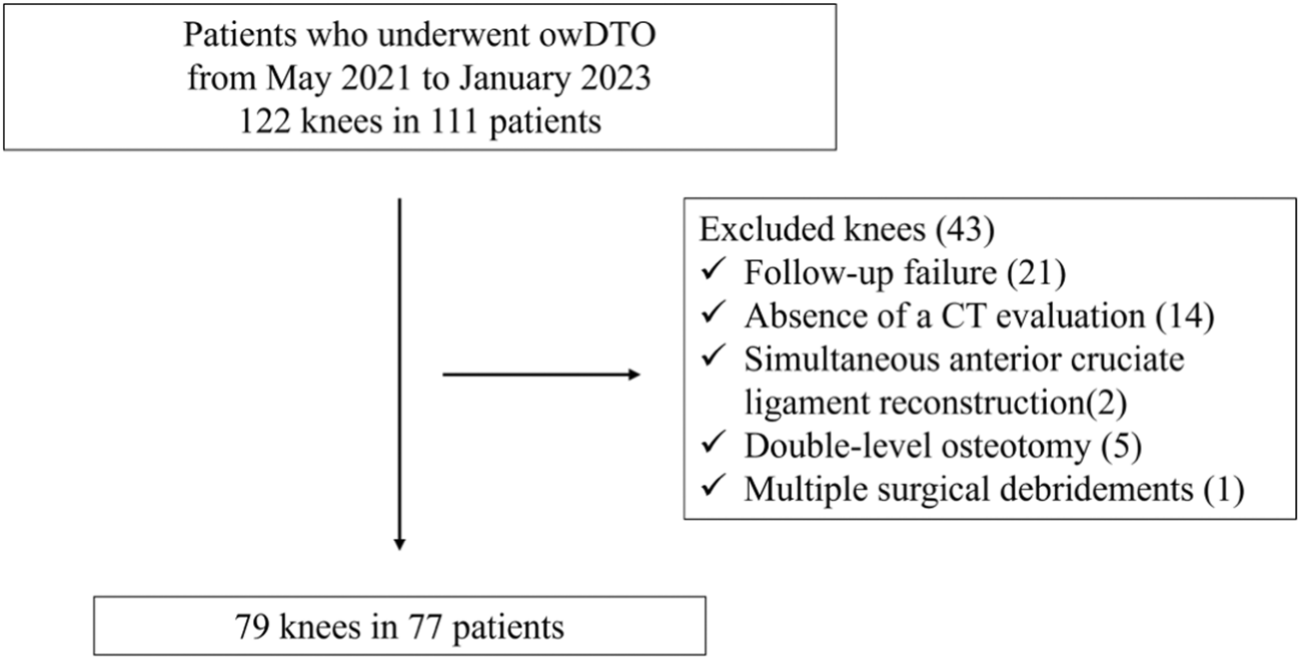
Flow diagram of patient selection. Patients who underwent open wedge distal tibial tuberosity osteotomy (owDTO) from May 2021 to January 2023 were included. Follow‐up failure, absence of a computed tomography (CT) evaluation, simultaneous anterior cruciate ligament reconstruction, double‐level osteotomy and cases requiring multiple surgical debridements for postoperative deep infection were excluded. The final study population consisted of 79 knees in 77 patients.

A total of 79 knees from 77 patients (43 males and 34 females; age, 55 ± 10 years) were analysed. Sixteen knees had MMPRTs, and 39 knees had other reparable tears. The exclusion criteria were failure to undergo a 6‐month postoperative follow‐up (*n* = 14), absence of CT evaluation at 6 months postoperatively (*n* = 21), simultaneous anterior cruciate ligament reconstruction (*n* = 2), double‐level osteotomy (*n* = 5) and cases requiring multiple surgical debridements for postoperative deep infection (*n* = 1) (Figure 3). All patients were followed for a minimum of 1 year, and bone union was ultimately confirmed in all cases.

Twenty‐two patients were current smokers. The mean BMI was 25.1 ± 3.8 kg/m^2^ (range, 17.9–34.9). LHF occurred in 20 knees overall, with 19 knees classified as Takeuchi Type I and 1 knee classified as Takeuchi Type II. The mean correction was 6.9 ± 1.5 mm (range, 4.0–11.0 mm). The mean P/D ratio was 0.6 ± 0.4 (range, 0.1–2.2). The distribution of KL grades was as follows: Grade 1, 5 knees; Grade 2, 49 knees; Grade 3, 15 knees and Grade 4, 10 knees. Preoperative and postoperative %MA were 34.6 ± 8.5% (range, 9–58) and 57.1 ± 5.8% (range, 43–74), respectively.

Bone union was observed in the posterior cortex, proximal flange and distal flange in 55 (69.6%), 76 (96.2%) and 67 (84.8%) knees, respectively (Table 1). Regarding the relationship between bone union at the distal flange and posterior cortex, the bone union rates for the posterior cortex were 77.6% in the distal flange union group and 25.0% in the delayed union group, respectively (odds ratio [OR]: 10.40, *p* < 0.01) (Table 2). The intra‐rater ICC was 0.80, and the inter‐rater ICC was 0.82 for the bone union of the posterior cortex, indicating good reliability.

**Table 1:** Number of bone unions and ratio in the posterior cortex, proximal and distal flange.

	Number of bone unions (*n* = 79) (%)
Posterior	
Zone 1	77 (97.5)
Zone 2	72 (91.1)
Zone 3	54 (68.4)
Zone 4	27 (34.2)
Zone 5	19 (24.1)
Most medial zone ≧ 3	55 (69.6)
Flange	
Proximal	76 (96.2)
Distal	67 (84.8)

**Table 2:** Relationship between bone union at the distal flange and the posterior cortex.

		Posterior cortex		Ratio (%)
		Delayed union	Union	
Distal flange	Delayed union	9	3	25.0%
	Union	15	52	77.6%

*Note*: Fisher's exact test, OR = 10.40, *p* < 0.01.

Abbreviation: OR, odds ratio.

The risk factors for delayed union of the posterior cortex and flange are summarized in Tables 3 and 4, respectively. Regarding delayed union in the posterior cortex, the rate of delayed union in the distal flange and LHF was significantly higher in the delayed union group than in the union group (delayed union group: 45.8%, union group: 16.4%; *p* = 0.01). Regarding delayed union of the distal flange, the percentage of smokers (delayed union group: 58.3%, union group: 22.4%; *p* = 0.03) and correction width (delayed union group: 7.7 ± 1.2 mm, union group: 6.8 ± 1.5 mm; *p* = 0.04) significantly influenced delayed union.

**Table 3:** Risk factors for delayed union in the posterior cortex: univariate analysis.

Variables	Union (*n* = 55)	Delayed union (*n* = 24)	*p* value
Age (years) (mean, SD, range)	53 (11, 24–78)	57 (9, 40–74)	0.12
Sex (*n* = 79)	32:23	11:13	0.33
BMI (kg/m^2^) (mean, SD, range)	24.9 (3.6, 17.9–34.0)	25.3 (4.5, 18.9–34.9)	0.67
Smoking (*n* = 22)	14	8	0.59
Flange distal union (%)	52.0	15.0	**<0.01**
LHF (*n* = 19: Type 1, 1: Type 2)	9	11	**0.01**
Correction width (mm) (mean, SD, range)	6.8 (1.5, 4.0–10.0)	7.3 (1.6, 4.5–11.0)	0.30
P/D ratio (mean, SD, range)	0.6 (0.4, 0.2–2.2)	0.7 (0.4, 0.1–1.7)	0.11

*Note*: Bold values indicate statistical significance at *p* < 0.05.

Abbreviations: BMI, body mass index; LHF, lateral hinge fracture; P/D, posterior‐to‐distal ratio; SD, standard deviation.

**Table 4:** Risk factors for delayed union in the distal flange: univariate analysis.

Variables	Union (*n* = 67)	Delayed union (*n* = 12)	*p* value
Age (years) (mean, SD, range)	55 (10, 24–78)	55 (12, 37–74)	0.95
Sex (*n* = 79)	35:32	8:4	0.53
BMI (kg/m^2^) (mean, SD, range)	24.8 (3.6, 17.9–34.9)	26.1 (4.9, 18.9–34.6)	0.30
Smoking (*n* = 22)	15	7	**0.03**
LHF (*n* = 20: Type 1 = 19, Type 2 = 1)	14	6	0.07
Correction width (mm) (mean, SD, range)	6.8 (1.5, 4.0–11.0)	7.7 (1.2, 6.0–10.0)	**0.04**
P/D (mean, SD, range)	0.6 (0.4, 0.1–2.2)	0.7 (0.5, 0.2–1.7)	0.47

*Note*: Bold values indicate statistical significance at *p* < 0.05.

Abbreviations: BMI, body mass index; LHF, lateral hinge fracture; P/D, posterior‐to‐distal ratio; SD, standard deviation.

Multivariate logistic regression analysis was performed to identify risk factors for delayed union in the posterior cortex and distal flange (Tables 5 and 6). Distal flange delayed union was significantly associated with the posterior cortex delayed union (OR: 12.70; 95% confidence interval [CI]: 2.42–67.20; *p* < 0.01). LHF was also significantly associated with lower odds of bone union (OR: 0.24; 95% CI: 0.07–0.86; *p* = 0.03). No significant associations were found with age, BMI or sex (*p* > 0.05). Regarding the distal flange, smoking was also identified as a significant factor associated with lower odds of bone union (OR: 0.15; 95% CI: 0.03–0.73; *p* = 0.02). No significant associations were found with age, BMI, sex, LHF or correction width (*p* > 0.05).

**Table 5:** Risk factors for delayed union in the posterior cortex: Multivariate analysis.

Variables	Odds ratio	95％ CI	*p* value
Age	0.95	0.89–1.01	0.12
BMI	1.03	0.89–1.20	0.70
Sex	1.89	0.57–6.27	0.30
Flange distal union	12.70	2.42–67.20	**<0.01**
LHF	0.24	0.07–0.86	**0.03**

*Note*: Bold values indicate statistical significance at *p* < 0.05.

Abbreviations: BMI, body mass index; CI, confidence interval; LHF, lateral hinge fracture.

**Table 6:** Risk factors for delayed union in the distal flange: multivariate analysis.

Variables	OR	95％ CI	*p* value
Age	0.97	0.89–1.05	0.43
BMI	0.96	0.81–1.14	0.67
Sex	0.84	0.18–3.93	0.83
Smoking	0.15	0.03–0.73	**0.02**
LHF	0.33	0.08–1.39	0.13
Correction width	0.69	0.42–1.13	0.14

*Note*: Bold value indicates statistical significance at *p* < 0.05.

Abbreviations: BMI, body mass index; CI, confidence interval; LHF, lateral hinge fracture; OR, odds ratio.

## DISCUSSION

The most important findings of this study were that smoking and LHF were associated with delayed union following owDTO. Specifically, smoking was associated with delayed union at the distal flange, whereas LHF was associated with delayed union at the posterior cortex. Although smoking has previously been identified as a risk factor for delayed union after open wedge HTO [3, 34], to the best of our knowledge, this is the first study to demonstrate an association between smoking and delayed union following owDTO.

In addition, our findings support previous reports identifying LHF as a risk factor for delayed union after open wedge HTO [4, 9, 18, 31]. Similarly, Ogawa et al. reported that postoperative‐onset LHF was associated with delayed union of the tibial tuberosity following owDTO [25]. Taken together, these findings suggest that LHF is an important risk factor for delayed union in owDTO and should be carefully considered during surgical planning and postoperative management.

In previous studies, an unstable LHF (Takeuchi Type II or III) was found to be a risk factor for delayed union in open wedge HTO [9]. However, in the present study, Takeuchi Type I was associated with delayed union. This difference may be attributed to the distinct osteotomy surfaces of open wedge HTO and owDTO. In open wedge HTO, the tension in the quadriceps muscle acts as a compressive force on the flange, whereas in owDTO, it exerts a force that separates the flange. This divergence may have compromised the stability between the proximal and distal fragments, even in cases of stable Takeuchi Type I LHFs, potentially leading to delayed union. In cases with LHF, bone union may be more dependent on the fusion of the posterior cortex; therefore, careful attention to posterior cortical healing is particularly important.

Distal flange delayed union was significantly associated with posterior cortex delayed union. In the present study, smoking was significantly associated with the delayed union of the distal flange. Although smoking was not directly detected as being related to bone union of the distal flange, it may be indirectly involved in posterior cortex union.

Correction width was not identified as a risk factor after multivariate analysis; however, there was a significant difference after univariate analysis. Since correction width has been reported as a risk factor for delayed union in previous studies [4, 29], it is possible that a larger number of cases may have yielded different results.

In the present study, the bone union rate of the owDTO group was 69.6%. Kobayashi et al. reported an open wedge HTO bone union rate of 83% using the same measurement method [16]. Another study demonstrated a radiographic union rate of approximately 90% 6 months after open wedge HTO [6]. Nejima et al. reported that owDTO may offer benefits in terms of bone union owing to the increased contact area of the flange [21]. A large trabecular surface area encourages revascularization and facilitates osteoblast migration, leading to bone healing [28]. However, in the present study, the bone union rate of owDTO at 6 months was lower than that of open wedge HTO, suggesting that the advantage of the contact area may be offset by other biological and mechanical factors. Further investigations are required to elucidate the mechanism of bone healing in owDTO.

This study has several limitations. First, although the post hoc power analysis indicated that the sample size was adequate, the number of patients included in the study was small. Further studies with larger sample sizes are warranted. Second, its retrospective single‐centre design may have resulted in selection bias and residual confounding. Third, evaluation at 6 months may be insufficient to assess the complete healing process. Although there is no consensus regarding the timing of bone union assessment, delayed union is commonly evaluated approximately 6 months postoperatively [20, 34]. Therefore, the timing of evaluation in the present study was considered clinically reasonable to evaluate delayed union. Fourth, clinical outcomes were not analysed. Therefore, correlation between delayed union and clinical outcome scores could not be assessed. Fifth, data regarding Vitamin D deficiency, rheumatologic disease and endocrine comorbidities, which could affect bone union, were not available in the present study. Sixth, detailed smoking information, including cigarette type, smoking frequency and previous smoking history, was not available. Therefore, the actual relationship between smoking exposure and delayed union may have been underestimated. Finally, some patients underwent concomitant meniscal or cartilage procedures at the time of osteotomy, but these factors were not included in the analysis. Despite these limitations, there are only limited reports on bone union in owDTO; therefore, understanding the union rate and modifiable factors that may influence it may be of significant clinical value.

## CONCLUSION

Smoking and LHF were associated with delayed union at 6 months in owDTO. Distal flange delayed union was significantly associated with posterior cortex delayed union. These findings may assist surgeons in identifying patients at risk for delayed union after owDTO and may contribute to improved postoperative management.

## AUTHOR CONTRIBUTIONS

Atsuki Tanaka and Ryo Okada contributed equally to this study and share first authorship. They were involved in the conception and design of the study; the acquisition, analysis and interpretation of the data; and writing the article. Daisuke Araki and Yuta Nakanishi were involved in the conception and design of the study, development of the research and writing of the manuscript. Takahiro Yamashita and Shohei Sano were involved in data acquisition and interpretation. All authors were involved in critical revisions of the article for their important intellectual content, and all approved the final version of the article.

## FUNDING INFORMATION

The authors have no funding to report.

## CONFLICT OF INTEREST STATEMENT

The authors declare no conflicts of interest.

## ETHICS STATEMENT

This retrospective study was performed in accordance with the Declaration of Helsinki and approved by our institutional review board (IRB No. 2306).

## Data Availability

The data generated and analysed during this study are included in this article.
